# Graphdiyne-Related Materials in Biomedical Applications and Their Potential in Peripheral Nerve Tissue Engineering

**DOI:** 10.34133/2022/9892526

**Published:** 2022-09-10

**Authors:** Xiao Li, Huiquan Jiang, Ning He, Wei-En Yuan, Yun Qian, Yuanming Ouyang

**Affiliations:** ^1^Department of Orthopedics, Shanghai Jiao Tong University Affiliated Sixth People's Hospital, Shanghai, China; ^2^College of Fisheries and Life Science, Shanghai Ocean University, Shanghai, China; ^3^Shanghai Engineering Research Center for Orthopaedic Material Innovation and Tissue Regeneration, China; ^4^Shanghai Eighth People's Hospital, Shanghai, China; ^5^Engineering Research Center of Cell & Therapeutic Antibody, Ministry of Education, School of Pharmacy, Shanghai Jiao Tong University, Shanghai, China

## Abstract

Graphdiyne (GDY) is a new member of the family of carbon-based nanomaterials with hybridized carbon atoms of sp and sp^2^, including *α*, *β*, *γ*, and (6,6,12)-GDY, which differ in their percentage of acetylene bonds. The unique structure of GDY provides many attractive features, such as uniformly distributed pores, highly *π*-conjugated structure, high thermal stability, low toxicity, biodegradability, large specific surface area, tunable electrical conductivity, and remarkable thermal conductivity. Therefore, GDY is widely used in energy storage, catalysis, and energy fields, in addition to biomedical fields, such as biosensing, cancer therapy, drug delivery, radiation protection, and tissue engineering. In this review, we first discuss the synthesis of GDY with different shapes, including nanotubes, nanowires, nanowalls, and nanosheets. Second, we present the research progress in the biomedical field in recent years, along with the biodegradability and biocompatibility of GDY based on the existing literature. Subsequently, we present recent research results on the use of nanomaterials in peripheral nerve regeneration (PNR). Based on the wide application of nanomaterials in PNR and the remarkable properties of GDY, we predict the prospects and current challenges of GDY-based materials for PNR.

## 1. Introduction

Elemental carbon plays an important role in supporting life on Earth. It is considered to be the most abundant element on Earth and has three different hybrid states: sp, sp^2^, and sp^3^ [1]. In the last two decades, many carbon-based nanomaterials composed of sp^2^- or sp^3^-hybridized carbon atoms have been discovered, including fullerenes, carbon nanotubes, graphene, and diamond, whereas acetylene bonds consisting of sp-hybridized carbon atoms have the advantage of linear and highly conjugated structures. Therefore, a long-term aim is to discover a new carbon isomer with sp-hybridized carbon atoms [2, 3].

In 1987, Baughman et al. predicted graphdiyne (GDY) to be a stable carbon material, which has sp and sp^2^-hybridized carbon atoms [4]. In 2010, GDY was first fabricated by Xie et al. through in situ crosscoupling [5]. Subsequently, researchers developed various synthesis methods, such as two-phase reactions and thermal coupling, which have achieved different GDY morphologies through continual optimization of catalyst systems, reaction classes, selection of templates, precursor structures, and other reaction conditions.

**Figure 1:**
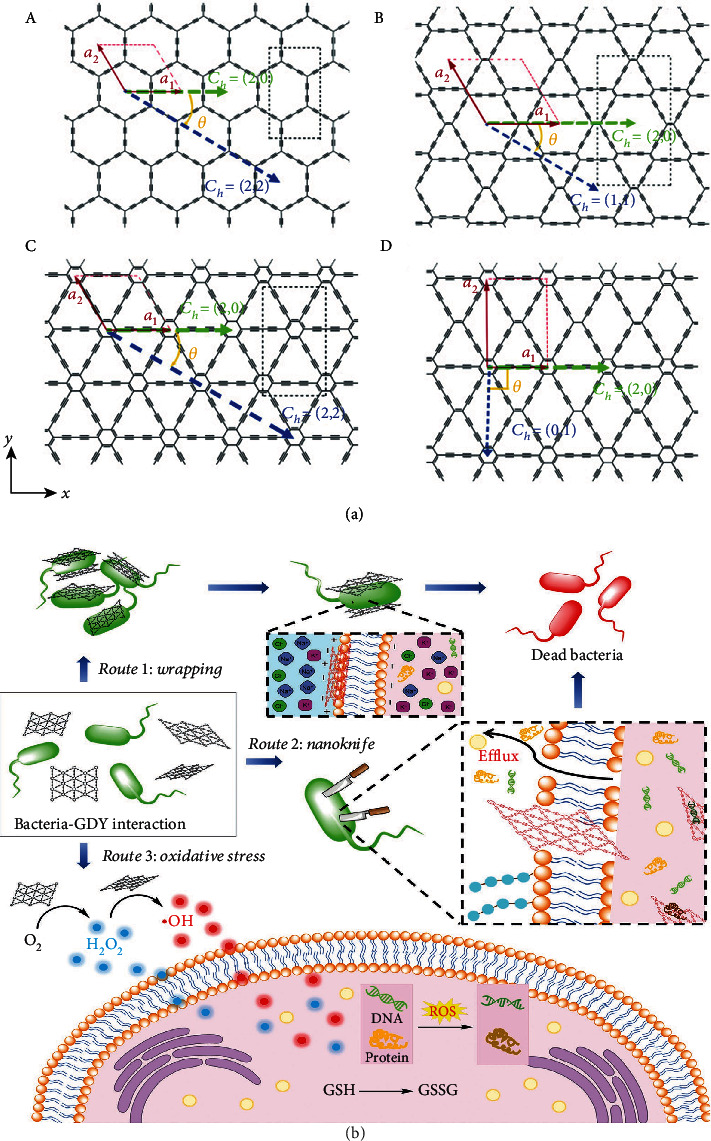
Structure of the different types of GDY and the possible collaborative antibacterial mechanisms of GDYs in terms of “physical” and “chemical” effects. (a) The different types of GDY. (A) *α*-GDY (B) *β*-GDY (C) *γ*-GDY, and (D) 6,6,18-GDY. Reproduced with permission from ref. [10]. Copyright 2020 Royal Society of Chemistry. (b) Schematic illustration of the possible collaborative antibacterial mechanisms of GDYs in terms of “physical” and “chemical” effects. Reproduced with permission from ref. [18]. Copyright 2020 John Wiley and Sons.

GDY is considered a new member of the family of carbon nanomaterials and includes *α*, *β*, *γ*, and (6,6,12)-GDY [6–8], as shown in Figure 1(a), which differ in the percentage of acetylene bonds they contain, where *α*-GDY is a conductor with the highest percentage of acetylene bonds (100%) [9], and *β*-, *γ*-, and 6,6,18-GDY sheets are semiconductors [10] containing 66.67%, 33.33%, and 41.67% acetylene bonds, respectively [9, 11].

GDY has been shown to have many advantages [11–13]. First, it has good mechanical properties, which are highly dependent on the presence of alkyne bonds. For example, as the percentage of alkyne bonds increases, the fracture stress of GDY decreases accordingly [11]. Secondly, GDY has excellent conductivity [12], where the intrinsic band gap of single-layer GDY is 0.44-1.47 eV, and the intrinsic electron mobility at room temperature is 10^5^ cm^2^ V^−1^ s^−1^, as determined by different calculations. In addition, the number of layers of the material also affects the electronic performance of layered GDY materials, and the most stable band gap for a double-layer and triple-layer GDY structure is 0.35 eV and 0.33 eV, respectively, while single-layer GDY has a higher band gap [14]. Furthermore, GDY has tailorable conductivity, making it suitable for various applications. Theoretical calculations revealed that the presence of acetylene bonds in GDY can effectively anchor various metal atoms to modulate their physicochemical properties [15]. The extraordinary nanostructure of GDY also provides highly active sites for adsorption of hydrogen atoms, while promoting proton reduction in electron-transfer processes, which provides it with unusual electrocatalytic properties. However, one study showed that hydrogen adsorption can degrade the mechanical properties of GDY [16, 17].

Furthermore, GDY has excellent antibacterial properties. Pan et al. revealed that CuO/GDY nanomaterials have high antibacterial activity with high inactivation rates for E. coli [13]. The antibacterial properties are thought to be multifaceted, including mechanisms related to various bacterial behaviors, such as encapsulation and insertion into the cell, which disrupts cell function and provides oxidative stress to the bacterial membrane. Figure 1(b) shows the possible mechanisms of antimicrobial activity of GDY-based nanomaterials in terms of physical and chemical effects [18]. In the case of the physical effects, when bacteria come into direct contact with GDY, encapsulation of the bacterial film may occur. Simultaneously, GDY nanosheets can be inserted into the bacterial cell membrane, leading to the loss of intracellular substances (e.g., proteins and K+). GDY may promote the generation of reactive oxygen species (ROS) via chemical processes and induce the metabolism of specific microbes. Furthermore, there are reports on the biosafety and degradability of GDY, but the findings are somewhat contradictory. Feng et al. showed that GDY can interfere with the structure of calmodulin, thereby affecting the regulatory function of calcium ions [19]. However, Liu et al. indicated that GDY has little effect on the structure of glucose oxidase and maintains high catalytic activity [20]. There have also been some studies on the biocompatibility of GDY using different cells. Wang et al. used murine-derived osteoblast-like MC3T3-E1 cells and showed that GDY promotes cell aggregation and adhesion [21]. Cao et al. used human umbilical vein endothelial cells to study the toxicity of GDY and graphite oxide [22]. They showed that GDY and graphite oxide were equally toxic, but at the same mass concentration, GDY was less inflammatory. M1 and M2 macrophages were also used to study the toxicity of GDY, and it was found that GDY produced little or no cytotoxicity. Further, it was proved that graphdyine oxide (GDYO) could be biodegraded through the NO/superoxide-peroxynitrite-driven pathway [23].

Finally, GDY has excellent properties, such as uniform pores, highly *π*-conjugated structure, high thermal stability, low toxicity, biodegradability, and fascinating conductivity [24]. Therefore, it has been widely studied in energy storage, catalysis, and energy fields, in addition to biomedical fields, such as biosensing, cancer therapy, drug delivery, and radiation protection [25]. Specifically, GDY shows great potential for peripheral nerve regeneration (PNR). Peripheral nerve injury is commonly caused by trauma, surgical complications, and birth defects and greatly affects the quality of life of patients [26, 27]. At present, autologous nerve transplantation is the gold standard treatment for PNR [28]. However, owing to the lack of donor nerves, the widespread application of this method is limited [29–31]. Nerve guide conduits are an artificial means of guiding axonal regrowth and are increasingly regarded as an alternative cure for auto-nerve grafting [30]; therefore, the choice of materials is an important factor for manufacturing a good nerve conduit. GDY has great potential in this field. The aim of this review is to improve the understanding of the valuable properties of this pioneering metamaterial in the tissue engineering community. Here, we review the research advances in GDY-based materials in biomedical fields. In view of the emerging applications of GDY materials in biomedicine, we summarize their biodegradability and biocompatibility based on existing research. Finally, we predict the prospects and current challenges of GDY materials in the field of PNR.

## 2. Synthesis of GDY Materials

**Figure 2:**
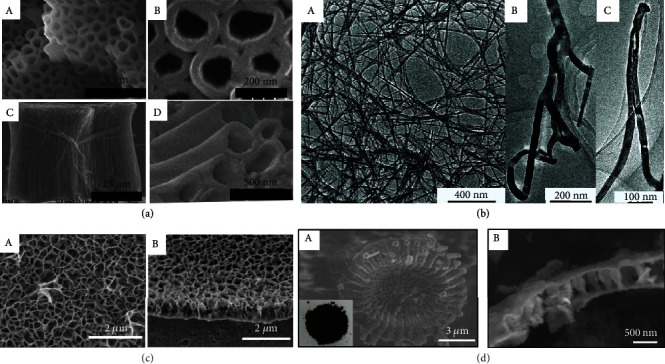
(a) The SEM images of GDNTs with different magnification. (A) Plan view. (B) Plan view under higher magnification. (C) End view. (D) End view under higher magnification. Reproduced with permission from ref. [35]. Copyright 2011 American Chemical Society. (b) TEM images of GDNWs under different magnifications. (A) TEM image under low magnification. (B, C) TEM images of high magnification (reproduced with permission from ref. [36]. Copyright 2012 The Royal Society of Chemistry. (c) SEM images of GDY nanowalls. (A) Top view. (B) Cross-sectional view. Reproduced with permission from ref. [38]. Copyright 2015 American Chemical Society. (d) SEM images of 3DGDY. Reproduced with permission from ref. [45]. Copyright 2018 John Wiley and Sons.

Typical synthesis techniques for GDY are classified as dry and wet chemistry [12]. Dry chemistry is mainly used to prepare materials on various substrates via gas–liquid solid-phase deposition. High-quality GDY has been obtained by dry chemistry, but large-scale production is limited by the insufficient preparation efficiency of GDY, which is mainly due to the rotation of carbon–carbon single bonds and the limited surface area of the substrate [32]. Wet chemistry is mainly carried out in solution, such as growth of carbon materials directly on copper sheets, or the use of growth based on the liquid–liquid or gas–liquid interface. Although wet chemical methods have the characteristics of low cost and ease of industrialization, the reaction process is difficult to control because of its high reaction activity. Therefore, both wet and dry approaches have their merits and limitations [32]. In 2010, Li et al. used copper foil as a reaction template for the crosscoupling of hexaethynylbenzene monomers and synthesized the first GDY film on this substrate with an area of 3.61 cm^2^ and semiconducting properties [33]. Subsequently, as shown in Figure 2, a wide variety of nanomaterials with different dimensions and morphologies of GDY have been prepared, and their excellent properties have been demonstrated. The morphology, structure, and dimensions of GDY are highly correlated with the preparation method and specific processes. GDY materials with different morphologies have been prepared by controlling the reaction conditions, GDY precursor, catalyst diffusion, morphology of substrates, and synthesis atmosphere [34].

### 2.1. Nanotube Structures

In 2011, Li et al. used anodic aluminum oxide attached to a template of copper foil, combined with a templating technique and catalytic crosscoupling reaction to prepare 15 nm-thick GDY nanotube arrays (Figure 2(a)), and showed superior field emission properties over carbon nanotubes and some semiconductors due to its high structural stability [35]. This material also had lower work function values than those of carbon nanotubes. GDY nanotube arrays have excellent field-emission properties, high chemical and physical stability, and good endurance to ion bombardment. Therefore, the novel molecular aggregation of GDY and its outstanding properties indicate its potential in the development of next-generation electronic and optoelectronic devices, particularly for vacuum device applications.

### 2.2. Nanowire Structures

In 2012, Qian et al. used ZnO nanorod arrays on silicon wafers as substrates for the successful preparation of aggregated structures of GDY nanowires, as shown in Figure 2(b), with lengths of 0.6–1.8 mm and diameters of 20–50 nm [36]. Electrical property measurements showed that the nanowire conductivity was 1.9103 sm^−1^, and mobility was 7.1 × 10^2^ cm^2^ V^−1^ s^−1^, which indicates excellent semiconducting behavior. Therefore, such materials have potential for efficient energy applications. It was shown that the synergistic effect between GDY nanowires and Cu is very important for the catalytic performance of an electrode [37]. The excellent catalytic activity and stability of a co-doped GDY hybrid structure were mainly attributed to the superactive metal substance, highly porous structure, high conductivity, and good chemical stability of GDY.

### 2.3. Nanowall Structures

**Figure 3:**
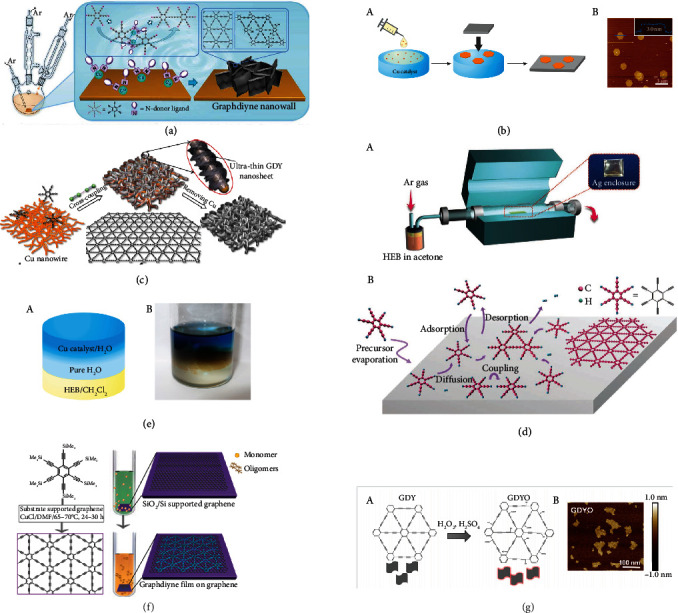
Schematic illustration of the generation of GDY with different methods. (a) Schematic illustration of the synthesis of GDY nanowall. Reproduced with permission from ref. [38]. Copyright 2015 American Chemical Society. (b) Schematic illustration of the production of GDY nanosheet via gas/liquid interfacial. Reproduced with permission from ref. [40]. Copyright 2017 American Chemical Society. (c) Representation of the synthesis of GDY nanosheet on CuNW paper. Reproduced with permission from ref. [41]. Copyright 2018 Wiley Oline Library. (d) Schematic illustration of the generation of GDY nanofilm with different methods. (A) Schematic illustration of producing GDY on the surface of the silver. (B) Schematic illustration of the surface growth process. Reproduced with permission from ref. [47]. Copyright 2017 John Wiley and Sons. (e) Schematic illustration of the synthesis of GDY through liquid/liquid interfacial. Reproduced with permission from ref. [40]. Copyright 2017 American Chemical Society. (f) Schematic illustration of the synthesis of GDY film on graphene. Reproduced with permission from ref. [49]. Copyright 2018 American Chemical Society. (g) The preparation process and morphology of GDYO nanosheets. (A) Schematic representation of the synthesis of GDYO nanosheets. (B) The morphology of GDYO nanosheets. Reproduced with permission from ref. [56]. Copyright 2021 American Chemical Society.

In 2015, Zhou et al. used hexaethynylbenzene as a precursor, along with pyridine, and small amounts of N,N,N,N-tetramethylethylenediamine and acetone as solvents and adjusted the reaction conditions of the Glaser–Hay coupling reaction to synthesize highly crystalline GDY nanowalls (Figure 2(c)) on copper foil (Figure 3(a)) [38]. These materials had superior field emission properties, porous structure, and specific surface area compared to graphene nanosheets, enabling them to be used directly as electrode materials for metal-ion batteries and provide abundant storage sites and channels, which imparted excellent electrochemical energy storage properties. GDY nanowalls provide abundant active sites for lithium storage for Li-ion batteries and act as effective pathways for fast ion diffusion. All of the above are attributed to the large specific surface area and hierarchical porous structure, with the presence of butadiyne linkages consisting of hybridized sp and sp^2^ carbon atoms. Therefore, GDY nanowires can be directly used as an electrode material for metal-ion batteries and capacitors [39].

### 2.4. Nanosheet Structures

In 2017, Matsuoka et al. were the first to synthesize GDY nanosheets (thickness of 24 nm) via a liquid–liquid interface method, as shown in Figure 3(e) [40]. Subsequently, the gas–liquid interface method was used to obtain regular hexagonal ~3 nm-thick multilayered GDY nanosheets (Figure 3(b)) [40]. In 2018, Shang et al. presented a new method for growing GDY nanosheets in which Cu nanowires were used as catalysts [41]. GDY nanosheets (thickness of 3.7 nm) were prepared (Figure 3(c)), which had high stability and excellent lithium storage properties. In 2021, Navaee et al. applied a new and powerful technique, whereby the bipolar electrochemical method was shown to be suitable for rapid high-yield synthesis of ultrathin GDY nanosheets with good photocatalytic activity [42]. GDY nanosheets have a porous structure and butadiyne linkages, and the number of active interfacial electrochemical sites can be increased to enhance fast ion diffusion, thereby optimizing the energy-storage capacity of the electrode material [43].

### 2.5. GDY Powder

The application of Cu substrates in the fabrication of GDY is limited because of their high cost and low surface area. In 2017, Zou et al. proposed an explosive method for the ultrafast generation of large quantities of GDY, in which hexaethynylbenzene precursors underwent crosscoupling reactions in the absence of a catalyst to synthesize three GDY powders with different morphologies. The resulting powder had excellent thermal stability, high conductivity (20 sm^−1^), and specific surface area (1150 m^2^/g), making it a bright prospect as a anode material for lithium/sodium-ion batteries [44]. In 2018, Li et al. successfully fabricated 3D GDY powder (Figure 2(d)) using naturally abundant and inexpensive diatomaceous earth as a template, which was used as a lithium-ion battery anode material owing to its appropriate structure, with high porosity and specific surface area [45]. GDY powder has an independent structure (no support material), large surface area, and high conductivity. In addition, the nonplanar structure prevents the GDY particles from stacking and agglomerating due to strong van der Waals forces and *π*–*π* interactions. The numerous pores provide many storage sites and transfer channels for lithium ions, which improves the electrochemical performance of GDY, making it suitable for use as a lithium-ion battery anode material.

### 2.6. Nanofilms

In 2015, Qian et al. used the method of reduction and autocatalytic gas–liquid–solid growth to fabricate GDY nanofilms of substrates of ZnO nanorod arrays [46]. The nanofilms had a conductivity up to 2800 scm^−1^ and field-effect mobility as high as 100 cm^2^ V^−1^ s^−1^; this was the first study to demonstrate the semiconductive behavior of GDY materials. In 2017, Liu et al. prepared large-area single-layer GDY films (conductivity of 6.72 scm^−1^) on silver foil via chemical vapor deposition of hexaethynylbenzene [47] (Figure 3(d)). In 2018, Zhang et al. fabricated two-dimensional ultrathin single-crystal GDY films on graphene substrates (Figure 3(f)) via solution-phase van der Waals epitaxy, which had high electrical conductivity (3180 Sm^−1^) and p-type semiconducting properties [48]. Furthermore, the fabricated GDY/graphene films showed great potential for use in NH_3_ gas sensors. In 2019, Liu et al. used graphene as a surface template to synthesize GDY films (thickness of ~2.9 nm), and the successful synthesis of GDY was attributed to strong *π*–*π* interactions between GDY and graphene [49]. The same method was used to obtain *β*-GDY films with smooth morphology and high crystallinity [50]. This template method could accelerate the development of ultrathin GDY films and provide broad prospects for their application in electronic devices. GDY nanofilms have high conductivity, large surface area, short diffusion distance, and a large number of active storage sites for metal ions. Therefore, they have the potential for use as electrode materials with high power and energy density.

### 2.7. GDY Quantum Dots

GDY quantum dots may have superior biological activity owing to the presence of active acetylene units. The classical solvothermal method was used to prepare GDY quantum dots with good biocompatibility in vivo and effective cell uptake and imaging functions [51]. Bai et al. prepared GDY quantum dots via an ultrasonic method for the first time and demonstrated their strong blue–green emission with a quantum yield of 14.6% [52]. The emission was effectively quenched by Fe^3+^ and recovered using ascorbic acid. Therefore, the quantum dots show great potential as a fluorescent nanosensor for detecting serum samples containing Fe^3+^ and ascorbic acid. However, the application of GDY is limited in many fields because of its poor solubility. Guo et al. prepared GDY-Py quantum dots (average diameter of ~3 ± 0.1 nm) via the Sonogashira crosscoupling reaction, which showed superior dispersibility in many organic solvents and water [53]. Moreover, GDY-Py quantum dots have excellent solubility, high stability, noncytotoxicity, bright fluorescence (quantum yield of 42.82%), and long fluorescence duration, thereby showing great potential in the fields of optical imaging and biomedicine. Therefore, owing to the size effects of GDY quantum dots, they possess many unique advantages, such as a band gap generated by quantum confinement, great dispersibility, abundant active sites, and good biocompatibility. Experiments have shown that GDY quantum dots have excellent light stability, can stimulate pH-dependent fluorescence emission, have effective cell uptake and cell imaging ability, and do not induce detectable cytotoxic effects in vitro. Therefore, they have great potential for application in biological imaging and other fields.

### 2.8. Synthesis of Graphdiyne Oxide

GDYO is usually synthesized from GDY powder via oxidation by H_2_O_2_ and H_2_SO_4_ [54–56], as shown in Figure 5. The use of strong oxidizing agents results in uncontrolled oxidation, and the GDY skeleton is often partially destroyed. Compared with GDY, GDYO has better biocompatibility and less cytotoxicity owing to abundant functional groups (such as carboxyl, hydroxyl, and epoxy groups) on the surface, which provides great potential for biomedical applications such as antibacterial and sensing functionality. Moreover, GDYO has certain potential as an electrode material. For example, Wang et al. developed a novel strategy for the surface modification of GDYO on Zn foil, which greatly improved the plating/stripping stability of Zn metal anodes since GDYO has high porosity with uniformly distributed pores, high hydrophilicity, strong coordination effect with Zn^2+^, strong *π*-conjunction, and unique semiconducting properties [57]. Therefore, GDYO is a promising surface modification material for Zn anodes toward the fabrication of full cells with ultralong cycling stability.

## 3. Applications in Biomedicine

**Figure 4:**
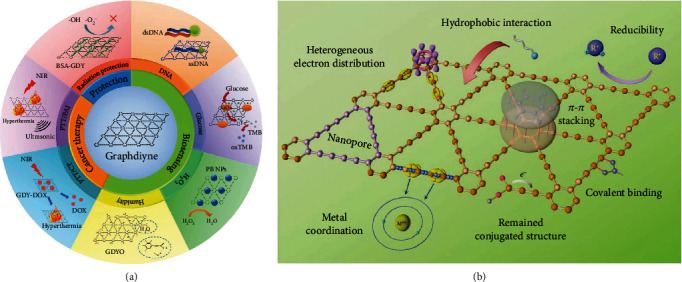
GDY application and advantages in the field of biomedicine. (a) GDY application in the field of biomedicine. (b) The advantages of GDY. Reproduced with permission from ref. [3]. Copyright 2019 John Wiley and Sons.

In recent years, GDY has attracted the attention of many researchers for use at the molecular level due to its advantages (Figure 4). In this section, we review biosensing, cancer therapy, drug delivery, and bioengineering applications, along with a brief description of the biosafety and degradation of GDY in biological environments.

### 3.1. Sensors

Owing to its acetylene bonds and abundant highly *π*-conjugated structure, GDY has a large adsorption energy for various substances and can be used in biosensors that benefit various economic sectors, such as environmental monitoring, food processing, pharmaceuticals, and clinical testing. Some representative examples of such sensing applications are discussed here.

#### 3.1.1. Humidity Detection

In industrial and agricultural production, environmental protection, and other sectors, the environmental humidity often needs to be measured and monitored. In recent years, it has been shown that GDYO is a potential humidity-sensing material because of the strong electron absorption performance due to sp-carbon hybridization, which accelerates its binding to water. Therefore, GDYO has a very fast response to humidity (~7 ms), and under the conditions of the same thickness and oxygen-to-carbon ratio, its response is three times faster than that of graphene oxide [58]. For carbon-based materials, humidity sensing is actually monitoring the conductivity change caused by water adsorption, which is owing to the formed hydrogen bonds between water and hydrophilic functional groups on the carbon [3].

#### 3.1.2. Drug Molecular Detection

Yuan and Mohamadi showed that the adhesion of the anticancer drug 5-fluorouracil to GDY sheets was poor; however, after doping GDY sheets with B atoms, the prominent importance of Q was observed, which effectively resulted in adsorption and charge transfer [59]. Compared to GDY, the adsorption energy of 5-fluorouracil was greatly increased for B-doped GDY, making it suitable for drug sensing. A study of the electronic properties of GDY and B-doped GDY sheets using density functional theory to monitor their adsorption and sensing ability toward temozolomide showed that GDY has only a very slight attraction to temozolomide drugs, while B doping significantly enhanced temozolomide drug adsorption [60]. Therefore, B doping of GDY is an effective strategy for enhancing drug adsorption and making this material more suitable for drug sensors.

#### 3.1.3. DNA Detection

DNA sensors can provide clinical disease diagnosis. As DNA is an important marker of human health, DNA sensing is of great importance. In recent years, based on the presence of sp-carbon hybridization in GDY, single-stranded DNA adsorption was facilitated, and it was demonstrated that GDY and GDYO can quench the fluorescence of organic dye-labelled single-stranded DNA probes via van der Waals forces and *π*–*π* stacking interactions between nucleobases and GDY. Hybridization with complementary DNA oligonucleotides creates double-stranded DNA, weakening the interactions between GDY and nucleobases, thereby liberating double-stranded DNA and ultimately restoring the fluorescence. This led to a further increase in fluorescence quenching. Therefore, GDY and GDYO have been widely studied in the field of fluorescence sensing. The fluorescence bursting ability of GDYO is higher than that of GDY, which can be used for highly sensitive selective detection of DNA and thrombin [61]. In addition, few-layer GDY nanosheets were shown for the first time to have high fluorescence bursting ability with different affinities for single-stranded and double-stranded DNA, which can be used for highly sensitive real-time fluorescence detection of DNA with detection limits as low as 25 × 10^−12^ m [62]. The biosensor showed the best performance in detecting multiplexed DNA compared to sensors based on graphene oxide and MoS_2_ nanomaterials.

#### 3.1.4. Biomarker Detection

There exists various key compounds that are involved in important biological functions in the body, such as the regulation of biological activity and catalytic reactions. Therefore, long-term in vivo monitoring of such compounds is beneficial for determining an individual's health status and diagnosing diseases in real time [63].

Amino acids play important roles in human health. An ab initio study of the adsorption of four amino acids on single-layer GDY/graphene showed that the adsorption energy of all four molecules on GDY was higher than that on graphene, implying that GDY has better potential for use in a two-dimensional amino-acid biosensor than graphene [64].

MicroRNA is an ideal candidate biomarker of almost all human disease. Mohamadi et al. used GDY loaded with Au nanoparticles as a photoactive material with good photoelectrochemical performance to prepare a biosensor for efficient and sensitive detection of microRNA [65].

Glucose is an important energy source for the human body, plays an important role in human health, and is particularly useful in the evaluation of diabetes [66]. GDY sheets have strong adsorption capacity for ferrous ions and glucose oxidase, thereby forming a complex that maintained high enzymatic activity that was successfully used in a one-step glucose assay [20].

Dopamine is associated with neuronal plasticity as a neurotransmitter in the human body and regulates various physiological functions of the central nervous system, such as learning and memory [67, 68]. Acidified GDY nanotubes and shortened and acidified multiwalled carbon nanotubes on glassy carbon electrodes were used to prepare a novel sensor with good performance for the detection of dopamine in biological fluids [69].

#### 3.1.5. Gas Detection

GDY and hemin were used in a sensor with an ultra-fast nitric oxide (NO) response time [70], which was used to monitor NO released by cancer cells and normal cells in real time. The adsorption behavior and catalytic process of the active materials play an important role, where conjugated sp-hybridized C atoms with abundant electrons are expected to be favorable for promoting the interaction between NO molecules and GDY. In addition, GDY disperses the hemin atoms to prevent their oxidation and aggregation, thus promoting the interaction and catalytic activity toward NO. In addition, when GDY was heat treated at 600°C for 2 h in Ar, it was able to detect low concentrations of NO selectively; therefore, it is an excellent sensing material with high sensitivity (250 ppb, *S* = 6.2%) and stability. GDY-600 showed typical p-type semiconductor characteristics during the sensor performance test. When exposed to air, oxygen molecules were adsorbed and ionized by electrons derived from GDY-600, forming reactive oxygen species. In a NO_2_ atmosphere, the oxidizing NO_2_ extracts electrons from GDY-600 and reacts with surface oxygen species to form an adsorbed state on the surface of GDY-600 owing to its strong electron-withdrawing property [71].

#### 3.1.6. Other Molecular Assays

Glutathione is an antioxidant that prevents damage to cellular components caused by oxidative stress in bacteria [72, 73]. The thiol group of glutathione can be oxidized to disulfide bonds to produce glutathione disulfide. Because glutathione is considered an indicator of cellular oxidative stress [74–76], it is an important biomarker. Wang et al. designed a PdFe/glutathione nanocomposite for detecting glutathione in bacteria, which can also efficiently consume glutathione for bacterial disinfection, thus promoting wound healing without significant toxicity both in vivo and in vitro [77]. Wang et al. prepared a Pd/GDYO nanocomposite for the first time and found that the material had significant peroxidase-mimicking activity; based on color changes in the Pd/GDYO-H_2_O_2_-3,3′,5,5′-tetramethylbenzidine reaction, a rapid detection method was established for glutathione in drugs [78].

### 3.2. Cancer Therapy

Cancer is one of the main causes of death and a major burden to global public health. Currently, the main treatments are chemotherapy and radiotherapy, but these are often ineffective and have many complications and side effects [79]. Therefore, the search for less toxic and highly effective treatment methods has become a focus of cancer treatment. In recent years, the minimally invasive and high spatial and temporal precision of phototherapy has made it a promising treatment method for cancer, especially photothermal therapy and photodynamic therapy [80]. Phototherapy agents absorb light and convert it into heat, in addition to generating reactive oxygen species, including singlet oxygen (^1^O^2^) that can lead to cell damage or even death [81]. GDY and its derivatives, which have high specific surface area and are rich in functional groups (epoxy, hydroxyl, and carboxyl groups), have been extensively studied in this field. GDY materials have been combined with anticancer drugs to form complexes through modifications and control of the particle size. The ability of GDY to cross the blood–brain barrier makes it suitable for targeted therapy with cancer drugs.

#### 3.2.1. Photothermal Therapy

**Figure 5:**
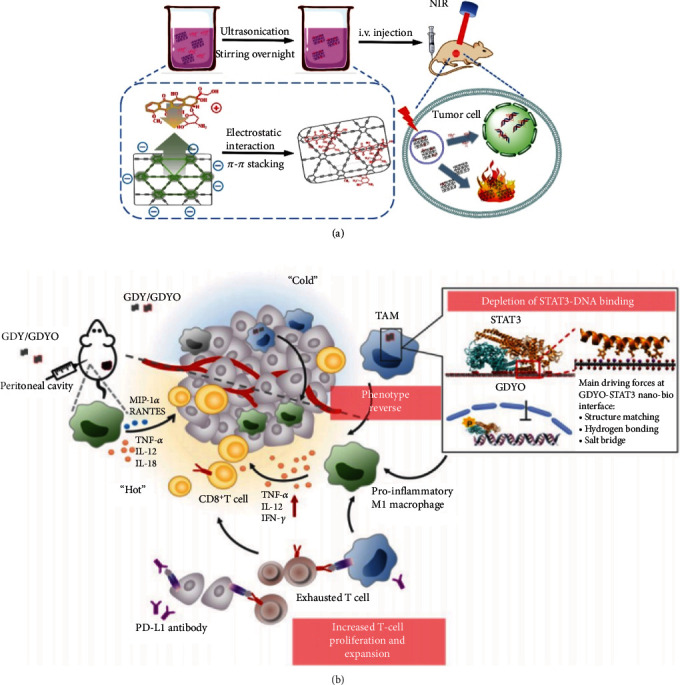
(a) The combination treatment of photothermal/chemotherapy for cancer. Reproduced with permission from ref. [85]. Copyright 2018 American Chemical Society. (b) Schematic illustration whereby GDY oxide nanosheets promote the polarization of macrophages. Reproduced with permission from ref. [56]. Copyright 2021 American Chemical Society.

Owing to its broad-spectrum absorption covering the entire visible light region, GDY has photothermal properties and can be used as an agent for photothermal therapy [82]. A composite based on GDYO with a high affinity for iron was demonstrated as an effective photothermal therapy agent (photothermal conversion efficiency of 37.5%), and the release of iron ions from the composite accelerated heat generation, thereby increasing the efficiency of the Fenton reaction and providing effective cancer treatment [83]. GDY-PEGylation was successfully applied for the first time in the photothermal therapy of mouse tumor cells, showing excellent photothermal performance (photothermal conversion efficiency up to 42%) [82]. Bionic ultrathin GDYO nanosheets with excellent photothermal conversion performance (efficiency of 60.8%) were shown to have sufficient overpotential for water oxidation and keeping a low band gap for absorbing red light; under laser irradiation (660 nm), the nanosheets produce cytotoxic singlet oxygen to relieve perfusion-limited hypoxia by inducing dilation of vessels and blood perfusion [84]. Doxorubicin was used as a model cancer drug and a live mouse photothermal/chemotherapy model combined with a drug delivery platform based on GDY nanosheets was developed [85]. As shown in Figure 5(a), both in vitro and in vivo treatments showed high cancer inhibition rates.

#### 3.2.2. Photodynamic Therapy

Photodynamic therapy is a new approach that generates reactive oxygen species in the presence of molecular oxygen to kill tumor cells [86–88]. Hypoxia often occurs in the core of solid tumors that are attributed to the abnormally fast proliferation of neoplastic cells. And neoplastic cells can produce a high concentration of H_2_O_2_ within solid tumors. Therefore, efficient conversion of H_2_O_2_ to O_2_ is a powerful way to overcome tumor hypoxia. GDY has high catalytic activity owing to its high surface area and sufficient sp-hybridized carbon that coordinates and bonds with metal atoms. So, GDY is a great template to form the metal/GDY composite due to many ultrasmall nanocatalyst molecules can be loaded and dispersed on the surface, and the formed composite possess high stability and persistent activity, generating O_2_ by decomposing H_2_O_2_.Therefore, GDY can be used to eliminate tumors in photodynamic therapy. GDY–CeO_2_ nanocomposites were formed by anchoring and dispersing cerium oxide nanoparticles on GDY; the composites showed superior catalase-mimicking activity, significantly alleviating tumor hypoxia, healing DNA damage caused by radiation, and inhibiting tumor growth [89]. Further, a composite was formed by immobilizing Pd nanoparticles (PdNPs) on the surface of GDY, where the resulting PdNP–GDY was used as an oxygen generator to reduce tumor hypoxia and inhibit tumor growth during long-term treatment in vivo [90]. Moreover, the combination of PdNP–GDY and doxorubicin showed a high antitumor efficiency of 82.9%. Ultrathin GDYO@i-RBM nanosheets effectively catalyzed the oxidation of water to produce O_2_ and further generate ^1^O^2^, showing excellent photothermal conversion efficiency of up to 60.8% in photodynamic therapy [84]. In 2021, Chen et al. demonstrated, for the first time, the interaction between GDYO nanosheets and intracellular protein coronae composed of signal translators and transcription 3 (STAT3) [56]. This interaction affects tumor-associated macrophage phenotypes, reversing them from a tumor-promoting M2 type to a tumor-suppressing M1 type, thereby enhancing immunosuppression in the tumor microenvironment; this mechanism provides a new strategy for immune-combined therapy (Figure 5(b)).

### 3.3. Drug Delivery

For local tumor recurrence and metastasis, effective therapies are required to eliminate residual tumor cells. Chemotherapy is the most commonly used method, but it is often accompanied by side effects [91–93]. Therefore, owing to the stability of drug-delivery systems and the controlled delivery effect, the local application of chemotherapeutic drugs is considered an effective solution to this problem. Nanomaterials with ultrahigh surface area can load various drugs and are being developed as drug carrier systems [94]. GDY has a high surface area and unique physicochemical properties and can load drugs effectively on the sheet surface mainly via *π*-*π* stacking and electrostatic interactions. The drug molecules are protonated, and the bond distance increases the drugs that are delivered to the target cell [85, 95]. Srimathi et al. proved that GDY nanotubes can be used as an effective drug-delivery system for chronic diseases by studying the properties of GDY nanotubes and the drug adsorption sites that protrude from the nanotubes [96]. It was reported that GDY nanosheets could effectively deliver quercetin and fluorouracil to target cells for the treatment of liver and colorectal cancers [95] and are also a good option for the delivery of flutamide drugs to treat prostate cancer [97]. Furthermore, GDY and micromotor technology were combined to inhibit the growth of cancer cells and control drug delivery [98]. The micromotor system has superior doxorubicin loading capacity (related to its unique GDY structure) and high biocompatibility compared to graphene micromotors.

In recent years, GDY composite materials have been demonstrated as drug carriers. Xue et al. fabricated a tumor-targeted drug delivery system Fe3O4@UiO-66-NH2/GDY to deliver the anticancer drug doxorubicin for tumor treatment [99]. This composite has many excellent properties, such as high biocompatibility, continuous drug release, and specific tumor-targeting capabilities. Furthermore, compared with traditional drug carriers, it has a higher delivery efficiency. As another example, a multifunctional nanodrug delivery system, GDYO-cisplatin/doxorubicon@DSPE-PEG-methotrexate, was used to treat cancer through a photochemical synergistic function [100]. The doxorubicin loading rate of GDYO-cisplatin was 40.3%, and its photothermal conversion efficiency (47%) and photodynamic effect were excellent under near-infrared irradiation. Some studies have also shown that electric fields and the introduction of other atoms can affect interactions between drugs and nanosheets. For example, when GDY was used to deliver idarubicin to cells, the structure and electronic properties of the drug molecules did not change significantly [101]. In addition, the application of electric fields in the *Z* and *XY* directions can enhance adsorption between drugs and nanosheets. In a theoretical study, density functional theory was used to clarify the interactions between an anticancer drug (hydroxyurea) and Gy and BNY nanosheets [102]. The results showed that doping of the Gy nanosheets with B and N atoms significantly improves the adsorption of hydroxyurea drugs. Therefore, BNY can be used as a promising delivery carrier for hydroxyurea. GDY-based nanomaterials used for cancer drug delivery are summarized in Table 1.

### 3.4. Radiation Protection

Nanomaterials have been proven to have many merits for radioprotection, including high efficiency, broad-spectrum free radical scavenging ability, good chemical stability in physiological environments, and long residence times in the body [103]. GDY has a strongly delocalized *π*-conjugated structure and highly reactive diacetylene linkages, which results in highly efficient and broad-spectrum radical scavenging activity [62, 104]. Therefore, GDY nanoparticles modified with bovine serum albumin (GDY-BSA) were studied for the first time for their radioprotective ability in the gastrointestinal tract. The results of in vitro experiments revealed that GDY-BSA effectively reduced DNA damage and promoted the vitality of gastrointestinal cells after radiation exposure. In vivo results showed that GDY-BSA could significantly reduce the discomfort caused by radiation in mice. Experiments also showed that GDY-BSA effectively inhibited the signaling pathway of apoptosis induced by reactive oxygen species, thereby reducing the apoptosis of gastrointestinal cells. These results are a good guide for nanomedicine in the treatment of gastrointestinal diseases [5]. Xie et al. developed a nano-GDY sodium hyaluronate hydrogel for the first time using GDY with powerful broad-spectrum radical scavenging activity and a high-water hydrogel with good low-energy X-ray attenuation ability; the experimental results showed that the hydrogel has good biosafety and can effectively reduce low-energy X-ray-induced skin oedema and ulceration in mice and alleviate pathological lesions, thus promoting wound recovery [105]. Ultraviolet light effectively kills cells. Yuan et al. showed that GDY has a unique optical nonlinear adsorption capacity and can absorb UV well owing to its sp hybrid structure and large *π*-conjugated system [106].

### 3.5. Wound Therapy

The skin plays an important role in the human body, as it is connects the internal and external parts of the organism and can prevent pathogen invasion. Inadequate treatment of skin damage can lead to disability or even death [107]. GDY shows great potential in this field owing to its excellent antibacterial properties and biocompatibility. For example, a hemin/GDY nanocomposite was used to catalyze hydrogen peroxide to generate highly toxic hydroxyl radicals via its high peroxidase activity [108]. Bacterial cell membranes are damaged by toxic substances, eventually resulting in bacterial death. Both in vitro and in vivo experimental results showed the biocompatibility of GDY-HM, which is suitable for the treatment of wound infections. PdFe/GDY nanocomposites have peroxidase-like activity and can efficiently catalyze the decomposition of H_2_O_2_ to produce •OH radicals. The excellent conductivity of GDY and the high local concentration of •OH induce tremendous oxidative stress, damage bacterial membranes, and eventually cause bacterial death. Furthermore, such nanocomposites efficiently consume glutathione for bacterial disinfection and promote wound healing without significant toxicity both in vivo or in vitro [77]. B-GDY has also been confirmed to contribute to wound healing by catalyzing the conversion of H_2_O_2_ to detrimental reactive oxygen species, such as •OH and •O^2−^ to eliminate bacteria by a POD-like mechanism; in addition, it had good biocompatibility and hemocompatibility [109].

### 3.6. Bioengineering

In view of the superior mechanical properties of GDY, such as its high elasticity and flexibility, its role in the field of bioengineering has been explored. In recent years, advances have been made, especially in the regeneration and differentiation of bone tissues and functional repair of the nervous system. Wang et al. described a GDY–TiO_2_ nanofiber composite and showed that GDY enhances the biocompatibility of TiO_2_ nanofibres [21]. In vitro, the composite can adsorb osteoinductive components, thereby improving cell proliferation and osteoinductive properties and further facilitating bone-tissue regeneration. Regarding the functional repair of the nervous system, a GDY-based artificial synapse was developed that can combine with presynaptic neurons to form a hybrid system, which can achieve synaptic plasticity and carry the biological signals [110]. However, the application of GDY in bioengineering is still in its infancy, and the use of advanced biotechnology to enable selective adhesion and directed growth and differentiation of GDY is worth exploring in the future.

## 4. Biosafety

**Figure 6:**
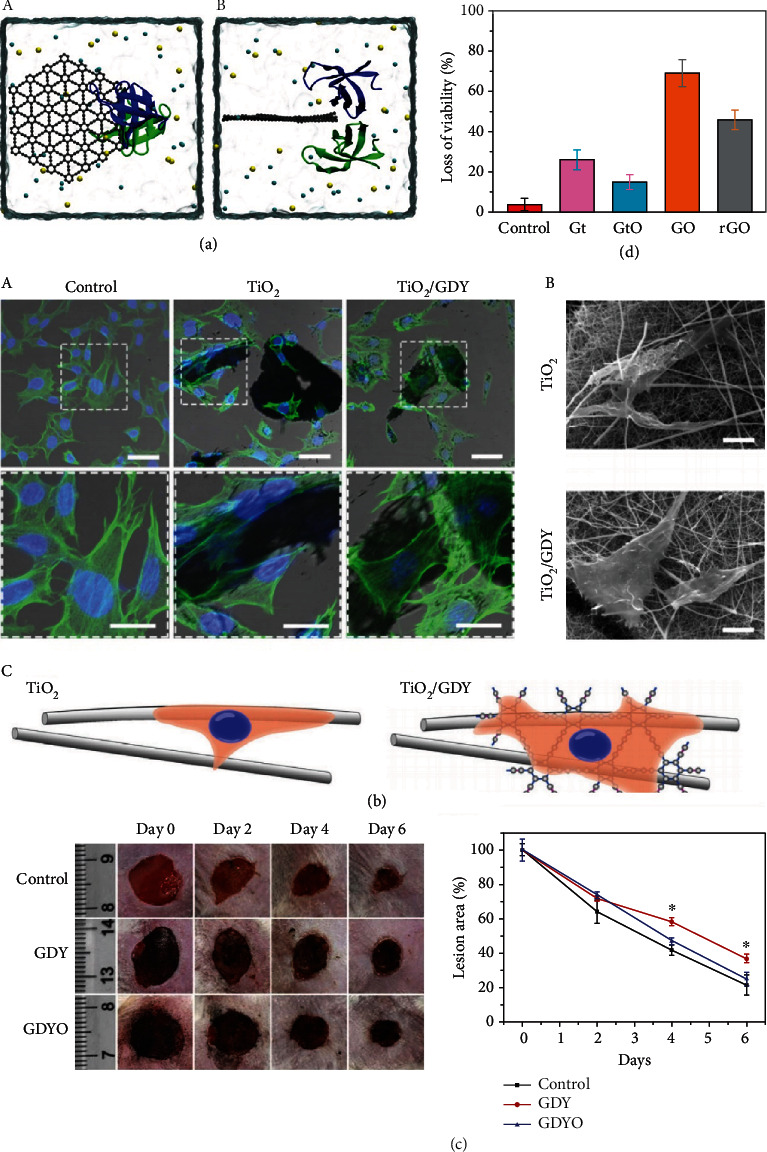
(a) Illustration of the simulation systems: two protein monomers of the dimer, colored in green and blue, respectively, sodium and chlorine ions, colored in yellow and cyan spheres. (A) Overhead view. (B) Lateral view. Reproduced with permission from ref. [111]. Copyright 2021 Royal Society of Chemistry. (b) MC3T3-E1 cell viability and adhesion of TiO2/GDY and TiO_2_. (A) Cytoskeleton staining of cells, phalloidin (green), and DAPI (blue). (B) SEM images of cell morphology on different nanofibers. (C) Cell adhesion on different nanofibers. Reproduced with permission from ref. [21]. Copyright 2020 Springer Nature. (c) The vivo cytotoxicity evaluation of GDY and GDYO. Reproduced with permission from ref. [18]. Copyright 2020 John Wiley and Sons. (d) Cell viability for Gt, GtO, GO, and rGO. Reproduced with permission from ref. [118]. Copyright 2011 American Chemical Society.

With the widespread use of GDY in biomedical applications, there is growing concern about the biosafety of GDY materials. Researchers have previously conducted toxicity studies on GDY. For example, the toxicity of GDY was investigated using large-scale all-atom molecular dynamic simulations with respect to their ability to interfere with protein–protein interactions [111]. The simulation results showed that GDY can indeed disrupt protein–protein interactions due to the presence of hydrophobic residues at the interface between GDY and the dimer (Figure 6(a)). Furthermore, calmodulin was used to test the behavior of GDY nanosheets, and it was shown that with significant structural interference of calmodulin by GDY, the inhibition of Ca^2+^ regulation may lead to a significant disruption of the calcium signaling pathway [19]. Thus, a potential molecular mechanism of GDY cytotoxicity was revealed: GDY hinders the structure and kinetics of the Ca^2+^-binding messenger protein calmodulin, leading to disruption of the Ca^2+^ signaling pathway. However, it has also been shown that GDY can easily adsorb glucose oxidase with minimal structural changes in the enzyme and maintain high catalytic activity [20]. In 2021, Wang et al. used murine-derived osteoblast-like MC3T3-E1 cells to assess the biocompatibility of TiO_2_/GDY during bone regeneration [21]. The results showed that cells on TiO_2_/GDY aggregated and adhered more readily than cells cultured on TiO_2_ alone, and the attached cells exhibited enhanced spreading and filamentous pseudopod extension (Figure 6(b)).

The use of GDY-based materials leads to contact between the materials and blood vessels. However, there are few studies on their toxicity. Cao et al. investigated the toxicity of GDY and graphene oxide (GO) on human umbilical vein endothelial cells [112]. Such cells are widely used to assess the toxicity of nanomaterials in vitro [113], as they are primary cells that can better prove the toxicity of nanomaterials toward normal cells. The toxicity of GO and GDY to human umbilical vein endothelial cells was comparable, but GDY showed less inflammation at the same mass concentration [22].

For GDYO, its unique structure and well-aligned groups of C=O and C-OH on the surface affect its interaction with biomolecules. A study showed that GDYO has a high affinity for proteins due to hydrogen bonding and salt bridges; so, GDYO can strongly interact with the activator of STAT3 and thus contribute to the polarization of M2 macrophages [56]. Macrophages play key roles in host defense, inflammation, and tissue homeostasis [114]. M1 macrophages mediate the responses to antibacterial and antitumor agents, whereas M2 macrophages are involved in tissue repair [115]. In addition, nanomaterials can modulate the polarization of macrophages, and the activation state of macrophages may determine the internalization of nanomaterials. A study of the toxicity of GDYO using macrophages showed that GDYO produced little or no cytotoxicity, and GDYO can reprogram macrophages from M2 to M1 [23]. It was confirmed that GDYO appears to be less cytotoxic and safer than GO [54] (Figure 6(c)).

Studies of the antibacterial performance of GDY and GDYO toward E. coli and S. aureus showed that the activity of GDYO was stronger than that of GDY against both bacteria, and the activity depended on visible light, bacterial species, and time [116, 117]. The antibacterial activity of GDYO was stronger toward S. aureus than E. coli under dark and visible-light conditions because of the difference in cytoarchitecture between Gram-positive and Gram-negative bacteria. In contrast, the activity toward E. coli is higher under visible light than under dark conditions [55]. In addition, the survival of both bacteria decreased significantly over time. However, several subsequent studies showed that at the same concentration, GO had the highest performance, followed by reduced GO, graphite, and graphite oxide [18, 118] (Figure 6(d)).

## 5. Biodegradability

**Figure 7:**
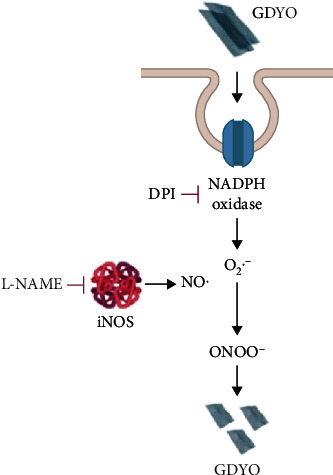
Schematic diagram showing the biodegradation process of GDYO. Reproduced with permission from ref. [23]. Copyright 2021 Royal Society of Chemistry.

Nanomaterials have biomedical applications, especially in tissue engineering. In addition to biocompatibility, biodegradability is equally important. An ideal tissue scaffold must be intact in the early stages to provide mechanical support for nerve growth. In the later stages, the scaffold should slowly be degraded or metabolized to provide sufficient growth space for nerve regeneration. Simultaneously, the scaffold should prevent reoperation and reduce inflammatory reactions to the stent/implant as a foreign body. Previous studies have shown that GO can be degraded by purified myeloperoxidase, which is released by activated human neutrophils [119, 120]. In addition, the dependence of peroxynitrite biodegradation on GO has been demonstrated [121]. A model of primary human macrophages was used to investigate the biodegradability of GDYO, as illustrated in Figure 7, showing that the biodegradation of GDYO occurs through the NO/superoxide-peroxynitrite-driven pathway [23].

## 6. Nanomaterials and Peripheral Nerve Regeneration

Nanotechnology offers new insights into innovative medicine, especially in tissue engineering, as carbon-based biomaterials can interact with tissues in various ways. Many studies have shown that the material properties (e.g., nanoscale topography and conductivity), material modification (e.g., loading with antioxidants or stem cells), and the preparation method (e.g., 3D printing and electrospinning [122]) all have a large influence on the growth and function of nerve cells.

First, because the fibrous structure of the scaffold can improve its performance and applications, the morphology of the scaffold surface has a significant influence on the growth and function of nerve cells. Common conduit structures include porous, multichannel, hollow, and grooved structures. A previous study showed that the surface of grooved fibers is rough, which is conducive to cell adhesion, and its orientation is conducive to axon growth, making such fibers suitable for use in tissue engineering [123]. Furthermore, proper methods are required to produce effective scaffolds. Compared with NGCs prepared by conventional methods such as electrostatic spinning, molding, and sheet rolling, more precise microstructures are obtained by 3D printing [124]. 3D printing was used to obtain a polydopamine/arginylglycylaspartic acid-coated graphene-loaded polycaprolactone nanoscaffold, which overcomes many problems, such as poor quality control, low mechanical strength, and uneven distribution of drug delivery [125].

Furthermore, material modification can improve the performance of the scaffold and promote PNR. Common modification methods include loading the surface of scaffolds with substances that promote cell adhesion, such as polydopamine [126] and arginine-glycine-aspartic acid [125]. In addition, modification methods include loading of stem cells (e.g., bone marrow mesenchymal stem cells and Schwann cells [125]) on the scaffold surface. Qian et al. prepared a polydopamine-coated gold/polycaprolactone nanoscaffold, where scaffold-loaded BMSCs and SCs were implanted into the body of rats, and showed that the scaffolds promoted cell adhesion, targeted transport of nutrients, and increased the expression level of CD31 to promote angiogenesis and repair of 15-mm sciatic nerve defects in rats [126]. In addition, modification methods include loading the scaffold surface with antioxidants such as melatonin [127–129] or (-)-epigallocatechin gallate [130]. Failure to regenerate axons is usually caused by severe energy shortage. Scaffold modification with bioactive antioxidants is a common approach for manipulating reactive oxygen species during PNR. Over the years, many scaffolds based on antioxidant modification have been demonstrated to improve the immune environment and promote PNR by reducing oxidative stress, inflammation, and mitochondrial dysfunction. Of course, many materials themselves also have antioxidant functionality, such as black phosphorus [131], nanoceramics [132], and nanodiamond [133], which can regulate the immune balance and effectively repair nerve damage by directional polarization of macrophages from M1 to M2 and reduction of persistent inflammatory responses.

**Figure 8:**
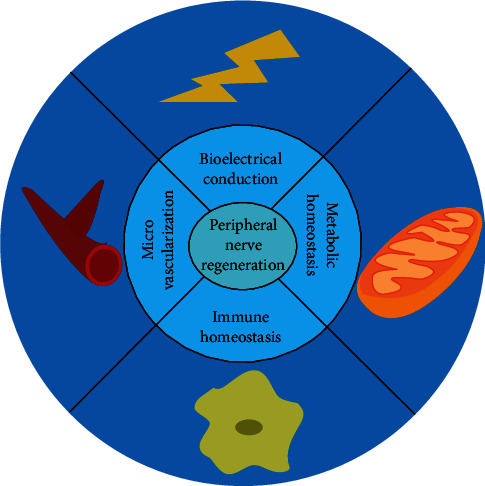
Summary of the four-factor microenvironmental cues during PNR.

Finally, the electrical conductivity of the material is also important for PNR, as conductive nanomaterials can directly and effectively repair nerve injury by restoring electrical conduction through electrical stimulation, because nerve tissues are electroactive. Thus, the recovery of bioelectricity signals is vital in PNR [134]. For example, our research group developed a series of new conductive nerve scaffolds, including those based on electrically conductive 3D graphene [125], GO [135], Au/polycaprolactone nerve conduit [126], and reduced GO/polycaprolactone [129], which can promote the remyelination of axons after nerve injury and restore bioelectrical signals to improve the efficacy of PNR. The GO nerve scaffold was also found to promote microvessel formation by activating the PI3K pathway [135]. In addition, we performed a preclinical evaluation of the graphene scaffold, and the results showed that the material could promote high-quality PNR without significant inflammatory response to local and surrounding tissues, providing evidence for the clinical transformation of graphene [136]. Subsequently, to overcome the problem that conductive materials need external electrical stimulation, we also developed a new piezoelectric nanoscaffolds, such as polycaprolactone composite scaffolds loaded with zinc oxide, boron nitride, or PVDF [137–139]. Under ultrasound stimulation, these materials can convert mechanical energy into electrical energy and achieve piezoelectric conductivity. Based on the above studies, we propose that four factors should be satisfied for nanomaterials to promote the PNR microenvironment: immune balance, microvascularization, metabolic homeostasis, and microelectrical conduction [140], as indicated in Figure 8.

## 7. Conclusions and Prospects

As a novel two-dimensional carbon allotrope, the specific structure and extraordinary properties of GDY and its derivatives make them attractive materials for various applications. The sp-hybridized carbon has a linear conformation that can enhance the pore size and provide sufficient sites for inserting other atoms, thus making it useful for ion-carrying and in situ catalysis, detection, and elimination of heavy metal ions and electrode materials for metal-ion batteries. GDY has been shown to be a suitable substrate for loading drugs, metal ions, proteins, and nanocatalysts because of its special alkyne bond and abundant highly *π*-conjugated structure. Therefore, GDY-based materials have been used in drug detection and delivery. The excellent photothermal conversion efficiency of GDY broadens its usefulness for tumor eradication by inducing hyperthermia. Therefore, it can be applied in photothermal therapy. GDY may coordinate with metal atoms and dock them onto the surface, resulting in a composite with high catalytic activity. Therefore, metal–GDY composites with high stability and persistent activity are useful for decomposing H_2_O_2_ and generating O_2_. Owing to these properties, it is widely used in photodynamic therapy. The capacity of GDY to scavenge reactive oxygen species and monitor humidity is enhanced by its sp-hybridized carbon atoms. GDY materials are also widely used in antibacterial, wound recovery, radiation protection, and sensing applications. However, there are still some challenges that need to be overcome. First, there are many challenges in obtaining high yields of high-quality GDY materials. Kong et al. reported a gap between the reality and ideality of GDY preparation [141]. Second, multilayered GDY nanosheets cover a portion of the acetylenic bonds, lowering the proportion of sp-hybridized carbon atoms and limiting the benefits of GDY. Third, owing to its unique sp-hybridized carbon structure, porous structure, large surface area, and excellent surface adhesion, GDY can specifically interact with biomolecules in a biological environment, but its nano-biointeraction mechanism is still not clear. Furthermore, although certain studies have suggested that GDY and its derivatives are safer than other carbon-based materials, the long-term biotoxicity of GDY is still unknown.

In the field of tissue engineering, research progress on materials from the same carbon family as GDY was discussed, such as the graphene/polycaprolactone conduit that can improve SC neural expression significantly in vitro, and promoted axon regeneration and myelin regeneration after nerve injury in vivo [125]. The GO/PCL scaffold promotes microvasculogenesis during PNR by activating the PI3K pathway [135]. The rGO/MLT/polycaprolactone scaffold was confirmed to promote PNR by improving mitochondrial function and restoring bioelectrical signals [129]. Therefore, based on the similarity of the material properties of the same carbon family (e.g., the excellent conductivity, low toxicity, and body degradability), we propose that GDY materials also have strong potential to promote PNR. In the future, mature preparation methods such as electrospinning [122], layer-by-layer casting, and integration molding could be used to prepare GDY nanoscaffolds with desirable morphologies, such as grooves and pores for drug loading. Such scaffolds could play an active role in the future of PNR.

However, the development of GDY materials is still at an early stage and faces many challenges. First, there is a major gap between the synthesis of ideal GDY and reality [141]. Parameters such as the size and number of layers of GDY and its derivatives have corresponding effects on the biological system, such as the strong interaction between GDY layers, which makes it difficult to obtain single-layer GDY sheets. Multilayer GDY sheets are undesirables because some of the alkyne bonds are covered by neighboring sheets, reducing the percentage of available sp-hybridized carbon atoms, and limiting the advantages of GDY. Therefore, exploring new synthesis strategies to reduce production costs and precisely control the properties of GDY requires further research.

Finally, the potential cytotoxicity and limited biodegradability of GDY require further investigation. This includes controlling the adsorption of nanoparticles to biomolecules in vivo once they enter the organism and reducing the toxicity of GDY through functionalization. The exact effect of GDY on cells, tissues, or organs and their metabolic pathways in vivo requires further elucidation. In vivo tracking of GDY remains a challenge that can be solved using various methods [142]. With the development of materials science, it is expected that GDY will be prepared with stable properties, well-defined structure, biosafety, and nontoxicity. Such achievements will broaden the application of GDY materials in the biomedical field, enabling them to enter into broader clinical research as a safe and effective medical material.

## Figures and Tables

**Table 1:** List of GDY-based nanomaterials used for cancer drug delivery.

Delivery system	Therapeutic agent	Type of disease	Drug loading rates	References
GDYO-@DSPE-PEG	Doxorubicin Cisplatin Methotrexate	/	40.3%	[100]
Fe3O4@UIO-66-NH2/GDY	Doxorubicin	/	43.8%	[99]
GDY nanotube	Flutamide	Prostate cancer	/	[97]
Graphdiyne micromotors	Doxorubicin	Kill HeLa cancer cells	/	[98]
BN analogue of GDY nanosheets	Hydroxyurea	/	/	[102]
GDY	Daunorubicin	/	/	[101]
GDY nanosheet	Quercetin, 5-fluorouracil	Hepatocellular carcinoma, colorectal carcinoma	/	[95]
GDY nanotube	Pentasa	Chronic disease	/	[96]

## Data Availability

The data of this study are available from the corresponding author upon request.
